# Nursing Home Residents Aged over 80—A Cross-Sectional Analysis on Which Activity Traits Correlate to Positive Affect

**DOI:** 10.3390/ijerph17249583

**Published:** 2020-12-21

**Authors:** Melanie Zirves, Holger Pfaff

**Affiliations:** 1 Gerontological Research on Well-Being—Graduate School GROW, Faculty of Human Sciences, University of Cologne, 50923 Cologne, Germany; 2 Institute of Medical Sociology, Health Services Research, and Rehabilitation Science (IMVR), Faculty of Medicine, University of Cologne, 50933 Cologne, Germany; holger.pfaff@uk-koeln.de

**Keywords:** affect, depressive symptoms, activity participation, nursing home, aged 80 and over

## Abstract

Admission to a care facility is assumed to enhance depressive symptoms and dependent behavior in old age. In this context, the relevance of participation in activities that make everyday life in a care facility more pleasant has been pointed out. This study examines if there is a relationship between participation in different activities as well as the frequency of this participation and the positive affect of nursing home residents aged over 80. Data from the unique cross-sectional representative study ‘Quality of life and subjective well-being of the very old in North Rhine-Westphalia’ in Germany (*n* = 150, aged 90.15 years in average) were used. The data were collected between 08/2017 and 02/2018 using computer-assisted personal interviewing. The variability in and frequency of activity participation functioned as independent, and positive affect as dependent variable. Multiple regression analysis was performed. Residents’ predicted positive affect significantly increased with a higher variability in activity participation. There was no independent effect of frequency in participation. Our findings indicate that there is a significant and positive relationship between participating in a high number of different activities and the overall positive affect of residents aged over 80 years. This does not hold true for the frequency of participation.

## 1. Introduction

The growing number of the oldest population (here defined as 80 years and over) might lead to an increased demand for inpatient care in the future [1]. Evidence shows that individual well-being is particularly influenced by change of living environment such as nursing home admission, preservation of independence, and social integration [1,2,3]. Furthermore, admission to a care facility is assumed to enhance depressive symptoms and dependent behavior in old age [2,3,4]. Both the prognosticated increasing number of nursing home inhabitants and the shown influence on the psychological well-being when moving into a nursing home also hold true for Germany [5,6].

One way to ease the process of adapting to a new living environment and to make everyday life in old age more pleasant is to participate in activities [1,7,8]. This also holds true for residents as has been shown by McGuinn and Mosher-Ashley [9] as well as Chao and Chen [10]. In this context, the relevance of activities that reduce stress, promote interest [11] and thus also allow the experience of one’s own competence and scope of action in institutions has been pointed out [5]. In this context, the frequency of participation in activities seems to play an important role [12]. At the same time, the variability in activities appears to be relevant [13].

In Germany and internationally, however, research on this topic is rare, especially regarding residents aged over 80 who are disproportionately overrepresented in nursing homes [5]. Hilleras et al. [14] investigated activity patterns of cognitively intact subjects aged 90 years and older. Silverstein and Parker [13] analyzed leisure activities and their impact on changes in quality of life in oldest-old Swedes. Chao and Chen [10] studied the role of activity profiles for very old adults in long-term care. Cho et al. [15] focused on positive and negative affect in oldest-old adults. No study could be identified that explicitly investigated residents’ participation in activities at an age higher than 80 years and no study was found dealing simultaneously with positive affect. In order to bridge this gap, we have analyzed if there is a relationship between the positive affect of residents and the two components of frequency and variability of activity participation in nursing homes.

### Conceptual Framework and State of Research

Following Lawton’s activity model (1983) [14], the antecedents and consequences of older adults’ participation in activities were distinguished in the current study. The antecedents comprise personal and competence variables (e.g., age, gender, education, activities of daily living (ADL)), preferences, and the environment (e.g., accessibility of the physical environment, activities offered). The consequences include personal meaning of activities, satisfaction, and psychological well-being [16,17]. In order to allow residents to continue their social participation and to maintain their quality of life, many nursing homes (NH) offer a variety of activities [1,5,18]. These activities should be freely selectable and ‘fit into a support process in which the primary purpose is to improve the fulfillment of daily life with satisfaction and enjoyment’ [1].

In the current analysis, activity is defined as ‘everyday activity NH-residents do as individuals, with their families and within their NH-community to occupy time and bring meaning and purpose to their life’ [19]. Measuring activities as well as categorizing them into different activity types represent challenges in research [20], but authors have confirmed that participation in different activities is more beneficial for quality of life than engagement in only one type of activity [10,13,21,22,23].

The extent to which offers of social [24], physical [25,26,27,28], and general activities [4,29,30,31] are related to depressive symptoms in long-term inpatients has been examined numerous times with diverging results. For instance, Roh et al. [32] have shown that a decrease in risk of depression in the elderly was associated with participation in physical, social, and religious activities, whereas Hsiao and Chen [2] could not observe any significant interaction between leisure activity participation and depression.

Chao [21] has pointed out that depressive symptoms consist of different components such as negative and positive affect, somatic symptoms, and interpersonal difficulties. Chao [21] is in favor of not analyzing summary depressive scores, but of performing domain specific analyses. Positive affect describes having an active, enthusiastic, as well as awake state of mind [33]. It refers to the experience of ‘many pleasant emotions and moods’ [34]. Lawton [15] has concluded in his model that depression in older people is related to both activity level and positive affect level. He has also pointed out that increasing participation in activities can help raise positive affect scores. Furthermore, Meeks et al. [35] have shown that activity engagement is associated with depression through its impact on positive affect. A number of other studies have also exhibited that there is a positive link between residents’ participation in specific activities and their positive affect [4,14,36,37,38]. This has led us to conduct an analysis of only one dimension of depressive symptoms: the positive affect. The objective of this study is to discover a potential relationship between the participation in activities and the frequency of this participation, related to the positive affect of nursing home residents aged over 80. Based on the literature review, we hypothesized: residents participating in several different activities and residents participating more frequently in activities declare a higher score of positive affect.

## 2. Materials and Methods

### 2.1. Data and Study Sample

Data from the representative cross-sectional study set of ‘Quality of Life and Well-Being of the Very Old in North Rhine Westphalia (Representative Study NRW80+)’ [39]—carried out between 08/2017 and 02/2018 in the most populated federal state in Germany (North Rhine-Westphalia (NRW))—were analyzed in this study. The federal state of North Rhine-Westphalia can be regarded structurally as a kind of mini Federal Republic of Germany. Germany itself can be described as average in terms of social structure in an international comparison. Furthermore, it has currently one of the fastest aging societies. With the 3-year study NRW80+ representative statements on the living conditions, quality of life and subjective well-being of very old people are made possible for the first time in Germany. A representative survey is being conducted to determine the circumstances under which very old people live, the role they play in our society and how they would like to live to be satisfied, even with various health impairments. The NRW80+ study was planned for many years. The study protocol, the designed questionnaire and the addressing of the very old were prepared by a feasibility study [39,40]. The standardized questionnaire as well as the data have been transmitted to GESIS and are publicly available [41].

Participants in NRW80+ were recruited drawing a random sample from the civil register including nursing home population. 8040 persons were approached, 1863 interviews could finally be realized. Among them were 150 residents of nursing homes. The latter were examined in the present study. Nursing homes here are defined as a type of residential care offering continuous nursing care, a safe environment, as well as physical and cognitive support in daily living activities [1].

Computer assisted personal interviewing (CAPI) of approximately 90 minutes’ length were conducted by the survey institute Kantar Public Germany at the respondents’ places of residence. The fact that the questionnaires used were standardized ensured concordance in the survey. As the oldest old persons and especially residents are difficult to reach and interview, proxy informants (relatives or nursing staff) were included when the target persons were unable to answer the questions for health reasons (acute illness or severe dementia).

### 2.2. Model Specification

The analysis in consideration of the research objective required the operationalization of seven constructs. Following Chao [21], depressive symptoms were operationalized in terms of positive affect. In NRW80+, a short version of the scale ‘Positive and Negative Affect Schedule (PANAS)’ [42] with five items (α = 0.89) was applied in the questionnaire to assess the positive feelings experienced within the past 12 months. Residents and proxies were asked: ‘How many times have you (has he / she) felt enthusiastic/ alerted/ elated/ inspired/ determined within the last year?’ All five items were answered on a five-step scale (1 = ‘never’ to 5 = ‘very often’).

In line with Lawton [15], personal (1) and competence-related indicators (2) were considered as independent variables in the current analysis. Personal factors (1) included age (1a), gender (1b), education (1c), residential attachment of participants (1d), and their participation in activities (1e). Education (1c) was operationalized in NRW80+ following the German aging Survey (DEAS) [43]. It was separated into the categories 1 = ’low’ (no vocational training; secondary school leaving or lower), 2 = ’medium’ (completed vocational training or university entrance qualification), and 3 = ’high’ (completed studies). Concerning residential attachment (1d), residents were asked: ‘How closely connected do you feel to your living environment?’ Answers ranged from 1 (‘not close at all’) to 4 (‘very close’).

Based on the theoretical background mentioned above, participation in activities (1e) was measured on two scales. Residents were asked in a first step: ‘Which of the following activities did you do within the last twelve months?’ The selection of the 17 activities that were probed was based on the survey of the Berlin Age Study I [44]. Just like in the Berlin Age Study [44] we asked for sports activities, participation in coffee parties, café visits, travelling, cinema visits, theatre or museum visits, artistic activities, hobbies, volunteering, playing games, and participation in further education or political events. Five questions were added to the items of the Berlin Age Study covering low-threshold activities in old age: taking a walk, receiving visitors, doing mental exercises, reading, watching television. This first scale had a width from 0 (‘No’) to 1 (‘Yes’). Thus, participants answered for every activity with ‘yes’ or ‘no’, depending on whether they participated or not. If the residents affirmed their participation in an activity, they were subsequently asked: ‘How often did you do this activity?’ The answers of this second scale ranged from 1 (‘daily’) to 5 (‘once a year’).

Afterwards, total scores (indexes) were calculated. The index for the participation in activities consisted of the mean value of the activities probed and shows a continuous width from 0 (participation in none of the activities probed) to 1 (participation in all of the 17 activities). Thus, higher values correspond to a higher heterogeneity and variety of exercised activities. The frequency index was composed of 16 items. Watching television was excluded due to the fact that it was recorded in hours. The width for frequency of participation was also continuous (from 1 = ‘daily’ to 5 = ‘once a year’) and can be interpreted with lower scale values indicating more frequent participation in activity.

Competence variables (2) comprised activities of daily living (ADL) measured according to Katz et al. [45]. ADL items focus, inter alia, on the ability to eat, dress, walk, or use the bathroom. The ADL scale values were calculated from the mean value of the respective seven items and interpreted on a continuous scale point width from 0 (‘Only possible with help’) to 2 (‘No help’). Higher values mean better functional ability.

### 2.3. Statistical Analysis

We conducted the statistical analysis with Stata 16.0 (2019) [46]. The first step was the descriptive analysis of all variables. Continuous variables are expressed as means with standard deviations and categorial variables are shown as percentages. Secondly, Spearman’s rho correlation was calculated. Finally, we conducted a multiple regression analysis to determine the effect of the number of activity participations as well as their frequency on the older individual’s positive affect. For this purpose, we calculated three models by taking into account different control variables: age, gender, education, functional health (ADL), and residential attachment. R^2^_adjusted_ was used as goodness of fit measure for the regression calculations.

Model I represents the full model and shows how the associations of the explanatory variables of frequency and variability appear under control of the others. Model II and III explore the interaction of the individual explanatory variables: Model II does not control for frequency of participation, model III excludes the variability.

Variables were tested for normality and homoscedasticity using the Shapiro-Wilk test and the Levene test. Multicollinearity was examined using the Variance Inflation Factor. Missing data were imputed for the regression analyses by multiple imputation. The dependent variable of positive affect showed 7 missings, while the frequency of participation showed 3 missings, and education showed 15 missings. The missing data does not seem to be intentional, related to outcome measurements or other variables in the study. Following the Markov chain Monte Carlo technique [47], 20 complete data sets using multivariate normal regression were generated. The results after multiple imputation and after complete case analysis allowed the same conclusions. This enabled us to use all possible information and to address the potential bias due to missing data [22,48]. Statistical significance was defined as *p* < 0.05. NRW80+ was approved by the Research Ethics Committee at the University of Cologne (Germany) (17-169) [39].

## 3. Results

150 people older than 80 years and living in a long-term care facility for 3.14 ± 0.291 years in mean were included. Table 1 descriptively shows participants’ characteristics. The study sample consisted of 111 women (74.0%) and 39 men (26.0%). Residents were aged 90.15 ± 0.40 years on average. 74.0% (*n* = 111) of the residents were directly available for the study, 26.0% (*n* = 39) weren’t able to participate. In these cases, a proxy was asked. Four proxies were spouses, 14 were residents’ children, eight were (professional) caregivers, and 13 were other relatives.

55.6% (*n* = 65) of the nursing homes were non-profit, 42.6% (*n* = 49) were privately operated. The size of the nursing homes ranged from 10 beds to 206 beds. More than half (55.6%; *n* = 79) of the residents described their residential attachment as very close or rather close.

Regarding the positive affect (Table 2), it was found that for all items, less than a third of the respondents answered the questions with ‘often’ or ‘very often’. About half of the respondents (52.9%, *n* = 74) never or rarely stated enthusiasm or were elated (48.0%, *n* = 68) within the past twelve months. Moreover, 47.0% (*n* = 64) never or rarely felt inspired, 47.4% (*n* = 62) never or rarely felt a determination, and more than a third (36.4%, *n* = 57) never or rarely felt alert.

Furthermore, 98.5% of the nursing home residents were regularly active in some form (46.7% (*n* = 69) participated in at least four different activities within the last twelve months, 34.0% (*n* = 49) in at least seven activities, 15.3% (*n* = 22) in at least ten activities). The average variability of activities was 0.30 ± 0.013 (range 0–1). This means residents engaged in five different activities on average. 40.7% (*n* = 61) have been physically active during the past twelve months and 19.3% (*n* = 29) of the residents go for a walk on a daily basis. Moreover, 32.0% (*n* = 48) were regularly mentally active, of which 35.4% (*n* = 17) did brain teasers on a daily, 45.8% (*n* = 22) on a weekly basis. 95.2% (*n* = 141) of the residents have received visits within the past year, of which 13.0% (*n* = 18) have received daily and 63.3% (*n* = 88) weekly visits. The average frequency of participation in activities was 2.31 ± 0.05 (range 1–5) with 84.4% (*n* = 123) of the residents taking part in the activities they mentioned at least once a week.

Table 3 shows the results for the Spearman correlations calculated in this study. Sample size for correlation analysis was *n* = 118 as we did not use multiple imputation at this step yet. The 32 missing values (*n* = 150–118) resulted from the different number of missings per variable included in the correlation analysis. The latter allowed the identification of initial trends as well as consistencies with the research literature. Using correlation analysis and the original sample an attempt was made to create a valuable basis for the following regression analysis.

A strong positive and statistically significant correlation was identified between the sum score of the PANAS scale and the variability in activities (r = 0.54; *p* < 0.001). A moderate correlation was found between PANAS and residential attachment (r = 0.32; *p* < 0.001). Furthermore, there was a moderate positive and statistically significant correlation between variability in activities and ADL (r = 0.28; *p* < 0.01) as well as residential attachment (r = 0.20; *p* < 0.05) and education (r = 0.25; *p* < 0.01). Age and education were moderately and positively correlated (r = 0.34, *p* < 0.001). The frequency of participation in activities showed no statistically significant correlations to any of the other variables.

Multiple regression analysis (Table 4) was performed to predict positive affect based on the variability and frequency of activities. This calculation intended to verify our findings from the correlation calculation and to answer our central research question which activity trait is more likely to predict positive affect. Sample size for regression analysis was 150 as we decided to use multiple imputation for this step. The results indicated that all of the models are significant predictors of positive affect (model I: F(7, 138.4) = 9.19, *p* < 0.001; model II: F(5, 140.4) = 7.55; model III: F(6, 139.5) = 8.18). We observed no multicollinearity in terms of VIF.

Model I best explains the relationship between positive affect and participation in activities (R^2^_adjusted_ = 0.30 ± 0.25). It includes both of the independent variables and all control variables. All of the models show significant and robust positive effects of variability on positive affect (β = 2.35–2.42; *p* < 0.001) as well as of residential attachment on positive affect (β = 0.25–0.27; *p* < 0.01). As was already apparent in the correlation calculations, the frequency of participation in activities is not significant in any of the models calculated.

## 4. Discussion

The present study focuses on one component of depression symptoms, as we have found strong arguments in previous studies which suggest that it is worth analyzing positive affect as a specific domain of depressive symptomology. This should allow not to overlook relevant information hidden under different facets of depressive symptoms [22,49].

The findings of the present analysis are in line with those of Silverstein and Parker [13] showing that a wider variety of activities is more beneficial for quality of life than only participating in a single type of activity. Silverstein and Parker [13] were able to demonstrate a cumulative effect of participating in different activities because each activity type makes its own contribution to different aspects of depressive symptoms. The data basis of this study did not allow for an investigation of such an accumulation as it was not possible to analyze the extent to which a single additional activity contributes to an increase in positive affect of a resident.

One the one hand, the fact that frequency of participation is not significantly related to an increase in positive affect scores contradicts the results of Pushkar et al. [12] as well as Meeks et al. [35]. On the other hand, our findings agree with those of Diegelmann et al. [30] as well as Hsu and Wright [24] assuming that not frequency alone, but the enjoyability of participating in activities counts for a decrease in depressive symptoms. It might be possible that enjoyability or meaningfulness function as mediating factors between positive affect scores and both participation in a high number of activities and frequency of participation. Further analyses should thus be carried out in this respect.

The model of successful aging established by Rowe and Kahn [50] assumes that an active engagement helps promoting positive mental health. Nevertheless, we have also shown that not only participation in activities influences the prediction of positive affect. As the literature points out, functional ability, reduced physical function [51,52], as well as environmental factors [2,17,53] are linked to depression in general or to positive affect specifically. It would also be conceivable that the phenomenon of frailty of nursing home residents plays a role in this regard [54]. In this study, ADL and residential attachment to the nursing home showed a significant correlation with positive affect. Residential attachment also showed significance in the regressions calculated. This is in line with the findings of Altinas et al. [18]. The analysis of other factors, such as frailty or environmental aspects such as the maintenance of social contacts, and their influence on both participation in activities and positive affect should be investigated in more detail in further studies.

Since the everyday life of nursing home residents is focused around health-related events, they are at risk of rarely having positive experiences and of having their negative affect deteriorate. The high incidence of depression in nursing homes might be attributed to these interrelations. Van Haitsma et al. [38] claim that positive affect is closely related to factors of the environment, whereas negative affect is rather associated with internal factors like health and personality. Altinas et al. [18] have proven that the number of activities a person engages in contributes to a better adjustment to the nursing home. With these results, Altinas et al. [18] as well as this study reproduce the findings of McGuinn and Mosher-Ashley [9]. Chao [21] has shown in a longitudinal analysis that an increase in social activities not only contributes to higher scores in positive affect, but also to a decrease in negative affect and interpersonal difficulties. Even though we have argued in favor of only analyzing positive affect as a specific domain of depressive symptoms, the link between depressiveness in general and the participation in activities can only be established if all specific domains of depression are considered in the analysis. Positive affect can thus be seen in close relation to other dimensions of depression. Many older adults, however, show depressive symptomatology but do not meet the criteria for a depression diagnosis [21]. For this reason, it is important to focus on the individual components of depressive symptoms, such as positive affect in this case.

NRW80+ did not study one particular facility, but residents came from many different facilities, spread across the federal state of NRW. This means that it was not possible to study one specific activity program. The fact that we asked for superordinate activities makes the surveyed activities comparable since it can be assumed that residents should have access to these activities regardless of the nursing home in which they live. Noting that activity engagement in long-term care settings seems to play an important role, activities on offer are increasingly seen as an indicator of quality of care [23,38]. Knowing about the attributing value of offering different activities in nursing homes for positive affect may be action-guiding for the nursing staff in how to carry out and offer activities. The current analysis is not limited to activities explicitly offered by the nursing home. It can be assumed that having the possibility to engage in activities outside of the nursing home has an influence on the feeling of independence while transitioning to a long-term care setting. Supporting activities outside of the nursing home can therefore also be an important task.

It should generally be noted that analyzing activity does not reveal a person’s lifestyle. However, counting participation in different types of activities, thereby showing the effect of variability, shows that nursing home staff should offer a series of different group and individual leisure activities. In this context, it is also important to identify a person’s interests and preferences and thus encourage continuous participation in activities that residents have enjoyed before moving into a nursing home. This is especially relevant for residents with physical or mental disabilities [10]. As Tak et al. [23] have shown, activities are especially interesting to residents if they are related to their previous work or life; age and cohort specific-experiences may be a factor. At this point, the importance of biographical work in nursing homes can be emphasized, in the context of which preferences could easily be identified. This goes along with the demand for person-centered care and staff’s engagement in offering activities to encourage participation. Meeks and Looney [31] have shown that staff behavior plays a central role for residents’ depressive symptoms in general and positive affect in particular. In this regard, governments or nursing home management should focus on interventions and make efforts to train staff.

As oldest-old persons and especially residents are difficult to reach and interview, proxy informants (relatives or nursing staff) were included in NRW80+ when the target persons were unable to answer the questions for health reasons. Although self-reports should always remain the gold standard [55], many studies have shown no significant differences between reports done by participants and proxies [15,56]. Perspectives of proxies might even be useful to gain different viewpoints on oldest-old subjective well-being [15]. Asking proxies (e.g., family or staff members) thus seems preferable compared to the alternative option of excluding those residents altogether from the analysis. Nevertheless, relatives and staff certainly do not always have the same estimation as those affected. This plays a role especially in the course of dementia [57,58]. As Crespo et al. [59] showed, assessments of proxies can underestimate the state of mind of residents. Ratings from residents about their own quality of life may be higher than those of proxies. Therefore, it must not be ignored that there may be a distortion.

### Strengths and Limitations

Our study offers two main strengths. Firstly, despite a small sample size, our analyses reveal significant results for a very specific group of individuals for which there is still little research. This emphasizes the importance of our results. By providing new insights our findings contribute to a better understanding of the activity engagement and positive affect of the oldest old population in Germany. Secondly, the data set represents a new and unique representative sample of nursing home residents with very good data quality. With this the results gain in significance beyond the German and European context.

The analysis has nevertheless some limitations. The sample size is relatively small considering the number of variables included. As some variables have shown missing values, we have decided to use multiple imputation to allow for a comprehensive analysis of the established hypotheses. Siddiqui [60] requires a minimum of 15 observations for each variable included. Exceeding the indicated minimal number of subjects per variable (seven in this study), this has been considered.

The cross-sectional design of our study prevents an analysis of cause-and-effect relations. As Janke et al. [61] have shown, it is also possible that residents with lower positive affect participate less in activities. Secondly, residential attachment may influence how activities in the nursing home are recognized. We therefore can’t prove if the effect is due to activity or residential attachment. The time spent in a nursing home might also have an influence on both participation in activities as well as well-being (regardless of participation in activities). We could not confirm this possible confounder effect in analyses that are not shown here. As we have dealt with a retrospective study based on the assessment of the residents on the one hand and on the evaluation of proxies on the other, it is possible that the Likert-type scale that was used for positive affect as well as the scales used for variability in activities and frequency of participation limit the precision of the answers given and thus may include recall bias. This may be accompanied by a memory bias in terms of remembering participation. Since very old people as well as their proxies were interviewed, it is conceivable that there are some imprecisions regarding the frequency of participation as well as the differentiation of the individual activities from each other.

The aim of the current analysis is to focus only on the associations between participation in activity and positive affect. Given the named limitations, our results are explorative and not fully conclusive, but bear in them the potential for future research. We stress that longitudinal analyses are indispensable to explore causal relationship between living environment, participation in activities, and positive affect outcome. In addition, multi-level models seem optimal in order to separate context features of facilities from effects of residential attachment and participation in activity. Furthermore, this would allow for approximate effects of nursing staff or nursing home management.

Whether or not nursing home residents can participate in activities depends on the availability of continuous and trained personnel to enable activation. It can be hypothesized and it should be investigated in further analyses if the human resource management has an impact on the degree of activation of nursing home residents. In line with this, it should be analyzed if the organization (the nursing home) as a whole has an influence on the human resource management and thus also on the emotional outcome of the residents.

## 5. Conclusions

Our findings support the hypothesis that participating in a high number of different activities is positively associated with the overall positive affect of residents aged over 80 years. In our study sample this effect was significant even after controlling for demographic characteristics, functional ability, and residential attachment. Frequency of participation was not significant. Our second hypothesis therefore must be rejected.

The present explorative study provides new data both on the German nursing home population as well as on the specific group of very old people. Although we did not use population-based analytical methods, the analysis provides new evidence on the potential associations between positive affect of nursing home residents aged over 80 years and both the engagement in different activities as well as the frequency of participating in activities. Our findings certainly warrant further investigation. Nevertheless, it can be assumed that our results are relevant for health policy as a whole and specifically for actors involved in long-term care, providing incentives for various policy or structural initiatives. Questions and analyses concerning aspects of depression in nursing homes—such as positive affect—indirectly represent an opportunity to involve nursing home residents in the development of quality indicators. Furthermore, surveys of activity preferences could lead to more individualized care and thus to greater satisfaction or well-being among residents.

## Figures and Tables

**Table 1:** Descriptive characteristics of study participants.

Variables	%	Mean	SD	Min	Max
Age (in years)	100 (*n* = 150)	90.15	±0.40	80	101
Women	74 (*n* = 111)	90.51	± 0.48	80	101
Men	26 (*n* = 39)	89.13	± 0.67	81	98
Marital status	100 (*n* = 150)			1	4
married	10 (*n* = 10)				
widowed	76.67 (*n* = 115)				
divorced	6.00 (*n* = 9)				
unmarried	7.33 (*n* = 11)				
Education	100 (*n* = 150)	1.67	±0.67	1	3
low	43.70 (*n* = 59)				
medium	45.19 (*n* = 61)				
high	11.11 (*n* = 15)				
Residential connection	100 (*n* = 142)	2.57	±0.09	1	4
Activities of daily living (ADL)	100 (*n* = 150)	0.98	±0.65	0	2

**Table 2:** Descriptive results concerning positive affect of the residents.

Expression	Mean	SD	Never	Rarely	Sometimes	Often	Very Often
enthusiastic (*n* = 140)	2.53	±1.20	23.57% (*n* = 33)	29.29% (*n* = 41)	23.57% (*n* = 33)	17.14% (*n* = 24)	6.43% (*n* = 9)
alert (*n* = 129)	2.95	±1.11	10.08 (*n* = 13)	26.36 (*n* = 34)	29.46 (*n* = 38)	26.36 (*n* = 34)	7.75 (*n* = 10)
elated (*n* = 142)	2.73	±1.12	12.68 (*n* = 18)	35.21 (*n* = 50)	24.65 (*n* = 35)	21.13 (*n* = 30)	6.34 (*n* = 9)
inspired (*n* = 136)	2.66	±1.12	15.44 (*n* = 21)	31.62 (*n* = 43)	29.41 (*n* = 40)	17.65 (*n* = 24)	5.88 (*n* = 8)
determined (*n* = 135)	2.64	±1.18	19.26 (*n* = 26)	28.15 (*n* = 38)	28.89 (*n* = 39)	16.30 (*n* = 22)	7.41 (*n* = 10)
Positive and negative affect schedule Sum Score (*n* = 143)	2.71	±0.08					

**Table 3:** Correlation calculation.

Variables	1	2	3	4	5	6	7
1 Age							
2 Sex	0.115						
3 Education	−0.094	−0.336 ***					
4 Activities of daily living (ADL)	0.090	−0.132	0.203				
5 Residential connection	−0.068	−0.015	0.175	0.116			
6 Variability in activities	−0.010	0.092	0.250 **	0.283 **	0.204 *		
7 Frequency of activities	0.114	−0.039	0.064	0.121	−0.126	0.051	
8 Positive and negative affect schedule (PANAS) Sum Score	−0.022	0.120	0.201 *	0.176	0.320 ***	0.544 ***	−0.143

*n* = 118; * *p* < 0.05, ** *p* < 0.01, *** *p* < 0.001.

**Table 4:** Regression analysis.

Variables	Model I		Model II		Model III	
PANAS	Coeff.	SD	Coeff.	SD	Coeff.	SD
Age	0.007	0.015	0.003	0.015	−0.050	0.016
Sex (male)	−0.247	0.178	−0.262	0.179	−0.445	0.188
Education	0.091	0.117	0.077	0.118	0.196	0.126
Activities of daily living (ADL)	0.083	0.120	0.097	0.122	0.328 **	0.116
Residential attachment	0.253 **	0.069	0.262 ***	0.070	0.276 ***	0.074
Variability in activities	2.423 ***	0.520	2.349 ***	0.524		
Frequency of activities	−0.222	0.120			−0.183	0.128
R^2^_adjusted (based on Fisher’s z transformation)_	0.302	0.251	0.286	0.245	0.193	0.166

PANAS: Positive and Negative Affect Schedule; *n* = 150; * *p* < 0.05, ** *p* < 0.01, *** *p* < 0.001.
